# Advanced Erosive Gouty Arthropathy of the Knee

**DOI:** 10.7759/cureus.51432

**Published:** 2024-01-01

**Authors:** Alexei Buruian, Susana Angelo, Emanuel Seiça, Fábio Fernandes, António Mendes

**Affiliations:** 1 Orthopaedics and Trauma, Hospital Distrital da Figueira da Foz, Figueira da Foz, PRT; 2 Orthopaedics, Hospital Distrital da Figueira da Foz, Figueira da Foz, PRT

**Keywords:** mssa bacteremia, knee osteoarthritis/ koa, infection, total knee, gout

## Abstract

A 57-year-old male, with chronic bilateral knee pain and a history of poorly controlled hyperuricemia leading to gouty attacks, underwent orthopedic assessment. Radiographic and MRI findings confirmed chronic gouty arthropathy with erosive bony defects, the most significant on the right proximal tibia. Total knee arthroplasty (TKA) was performed without any complications, addressing the bony defect with cement and a semi-constrained prosthesis. However, a gouty attack led to prolonged wound discharge and periprosthetic infection postoperatively, prompting revision surgery with debridement, antibiotics, and implant retention (DAIR). Intraoperative cultures revealed methicillin-sensitive Staphylococcus aureus (MSSA). The treatment included vancomycin and rifampicin. Two years post-surgery, the patient walked pain-free with a knee range of motion of 0-90º. This report highlights the complexity of treating gout-related knee osteoarthritis, emphasizing early intervention to mitigate risks of extensive surgical procedures and infections.

## Introduction

Gout is a common inflammatory disease characterized by the deposition of monosodium urate crystals in joints and tissues. If left untreated, chronic gout can cause internal derangements of the knee, and a few cases of the condition have been described in the literature [1-25]. In this report, we present a case of a patient with multiple intraarticular and intraosseous tophi revealed by MRI who underwent primary total knee arthroplasty (TKA) and subsequent revision surgery with debridement, antibiotics, and implant retention (DAIR) due to periprosthetic infection.

## Case presentation

**Figure 1:**
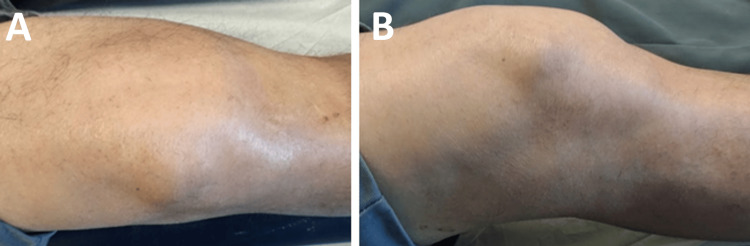
Clinical images of the right knee Coronal view (A), sagittal view (B)

A 57-year-old male was referred to the orthopedics department for the evaluation and management of persistent, chronic bilateral knee pain, with the right knee being more symptomatic. His medical history included hypertension, obesity, alcoholism, and poorly controlled hyperuricemia, resulting in intermittent gouty attacks over the past eight years and treated with colchicine during crises and no other medications. Clinical examination of the right knee revealed a limited range of motion (20-100º), joint line tenderness and crepitus, without any joint effusion or ligament instability (Figure 1).

**Figure 2:**
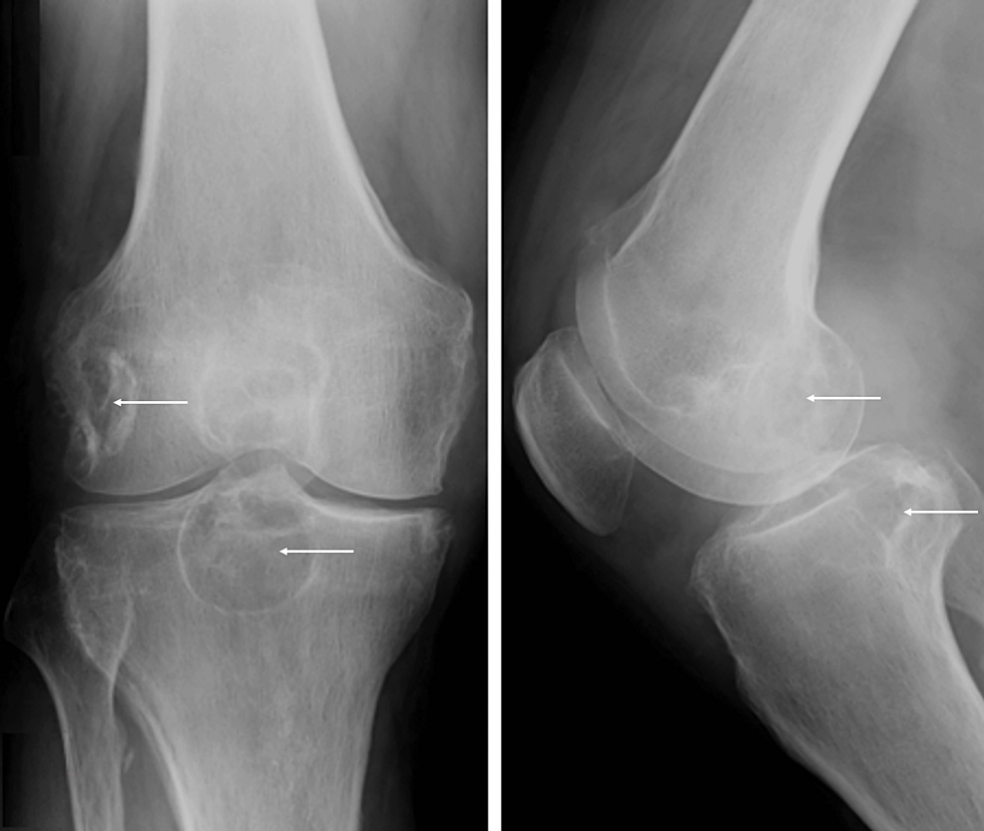
Radiographic images of the right knee Coronal view (A), sagittal view (B). The arrows point to the cystic formations in the proximal tibia and lateral distal femoral condyle

Laboratory analysis indicated elevated serum uric acid levels at 6.1 mg/dL (normal level: <7 mg/dL) during the consultation, with values as high as 12.4 mg/dL documented in previous flares. Radiographic images of the right knee displayed cystic formations in the proximal tibia and lateral distal femoral condyle, along with reduced medial joint space, subchondral sclerosis, and osteophyte formation (Figure 2).

**Figure 3:**
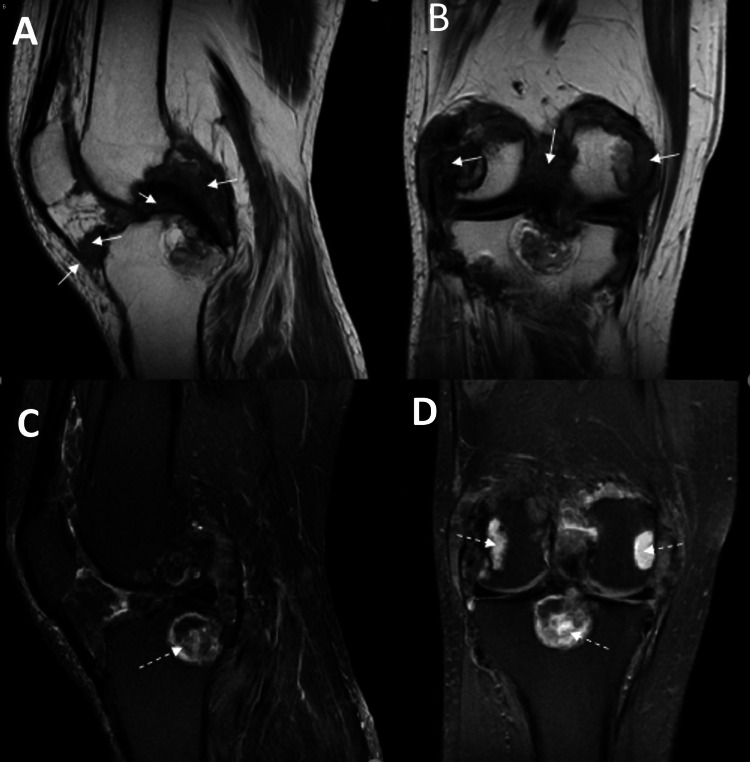
MRI of the right knee T2-weighted sagittal view (A), coronal view (B). T1-weighted sagittal view (C), coronal view (D). The arrows point to the location of the intra-articular tophi MRI: magnetic resonance imaging

Sagittal and coronal T1- and T2-weighted MRI scans confirmed chronic gouty arthropathy, revealing cystic changes suggestive of intraosseous tophi in both knees (Figure 3).

**Figure 4:**
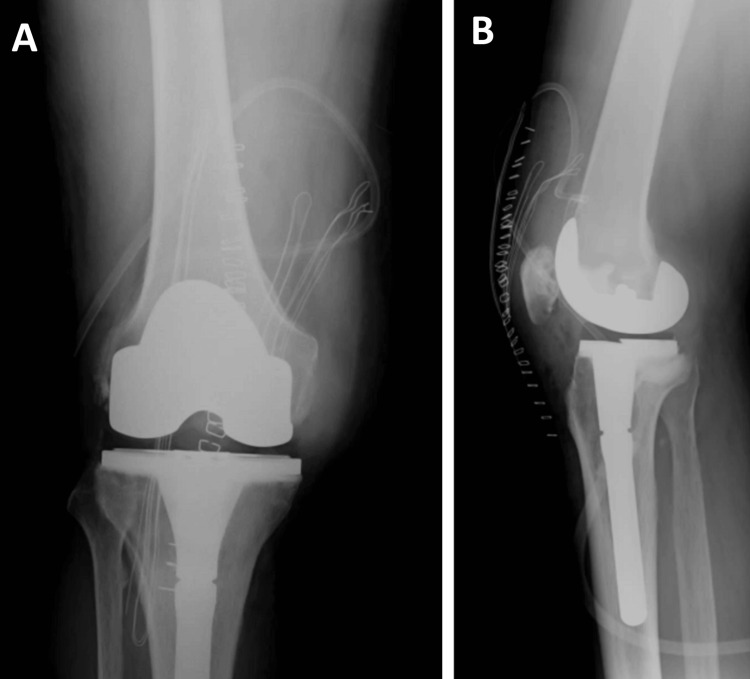
Postoperative radiography of the right knee Coronal view (A), sagittal view (B)

Given persistent knee pain, limited range of motion, and impaired daily activities, the patient underwent TKA without any complications. The surgical approach involved removing the visible tophi and filling the bony defect in the tibia with cement. A semi-constrained, cemented prosthesis was utilized, incorporating a stemmed tibial component due to concerns relating to potential ligamentous instability (Figure 4).

On the fourth day postoperatively, the patient exhibited sudden-onset severe pain, fever (38.1 ºC), increased local warmth, erythema, and edema, accompanied by a chalky white discharge from the wound, suggestive of a gouty attack. He was started on treatment with allopurinol and colchicine, experiencing notable improvement within three days. However, colchicine was discontinued due to diarrhea, and the patient was transitioned to a nonsteroidal anti-inflammatory drug (diclofenac). Despite initial progress, he continued to experience persistent drainage. By the 15th day postoperatively, significant wound closure had occurred, except for a communicating fistula with the prosthesis at the lower third of the wound. Laboratory results revealed elevated C-reactive protein (CRP) levels at 227.66 mg/L (normal value: <5 mg/L), an erythrocyte sedimentation rate (ESR) of 73 mm/1 h (normal value: <13 mm/1 h), and leukocytosis measuring 12.34 x 10^3^/uL (normal range: 4.0-10.5 x 10^3^/uL), with a neutrophil shift of 80.9% (normal range: 40-60%). 

The chalky material was not subjected to polarized microscopy or preoperative culture as it would not alter the clinical decision to perform a DAIR procedure. Two weeks later, the surgical wound closed without further drainage. Intraoperative culture revealed methicillin-sensitive Staphylococcus aureus (MSSA). The cystic and synovial lesion was sent for pathological examination, revealing a nodular formation filled with friable yellow material, accompanied by deposits of eosinophilic and fibrillar material, surrounded by giant cell reactions suggestive of a foreign body type. The diagnosis indicated chronic gouty arthropathy. The patient completed a therapeutic plan involving vancomycin and rifampicin, with inflammatory markers becoming negative at 12 weeks postoperatively, and was referred for medical consultation to manage and regulate serum uric acid levels. Two years post-surgery, the patient walked without pain, had a knee range of motion of 0-90º, and showed no signs of infection.

## Discussion

Gout is a highly prevalent condition, affecting around 3.9% of the American population, and is associated with a higher risk of knee osteoarthritis. Teng et al. reported a three-fold increased risk of knee osteoarthritis in knees affected by a gout attack, perhaps linked to an increased rate of cartilage wear related to soluble urate or urate crystals [26]. Gout is also a risk factor for complications following TKA and increased healthcare resource utilization. A study by Singh et al. evaluated National Inpatient Sample Registry data of discharges from US community hospitals and reported that gout exhibited a statistically significant association with elevated odds ratios (OR) for the following outcomes - discharge to a non-home setting: 1.18 (95% CI: 1.15 to 1.20); a hospital stay exceeding the median of three days: 1.08 (95% CI: 1.06 to 1.10); and transfusion, 1.15 (95% CI: 1.12 to 1.18) [27]. On the day of surgery and during the 90 days following the procedure, individuals with gout incurred significantly higher costs in comparison to those without the condition. A multivariate analysis has shown that patients with gout had a higher likelihood of developing medical complications compared to non-gout patients, especially in terms of wound-healing problems (12.2% vs. 5.0%) and renal complications (8.9% vs. 3.1%).

Relating to our study, the infection rate described was as follows: 6.85% of patients experienced wound drainage necessitating oral antibiotics; 2.69% had superficial wound infections treated with oral antibiotics; 1.04% of patients developed cellulitis requiring intravenous antibiotics; 0.62% of patients had cellulitis managed with oral antibiotics; 0.62% of patients had persistent wound drainage necessitating intravenous antibiotics; 0.21% of patient underwent surgical irrigation and debridement due to hematoma; and 0.21% of patients experienced partial skin necrosis around the surgical wound, which resolved with local wound care [28]. Rosas et al. aimed to evaluate the outcomes in gout patients undergoing TKA compared to non-gout controls. With a case-control design involving 15,238 Medicare patients, cohorts were matched based on age, gender, and the Charlson Comorbidity Index. The results indicated that gout patients incurred significantly higher day-of-surgery and 90-day post-surgery costs than the control group. Multivariate analysis demonstrated increased odds of complications for gout patients, including infection (OR: 1.229), cardiac arrest (OR: 1.354), pneumonia (OR: 1.161), hematoma (OR: 1.204), and capsulitis (OR: 1.208). Notably, gout patients had a decreased risk of pulmonary emboli (OR: 0.835). These findings support the hypothesis that gout patients undergoing TKA face elevated postoperative complication rates and increased costs [29].

To further complicate matters, the differential diagnosis between a gouty flare and periprosthetic knee infection is not straightforward and both can coexist, with the latter necessitating revision surgery [30-31]. Manifestations of gout include sudden onset of pain, redness, swelling, and increased warmth in the affected joint, which can resemble septic arthritis. A conclusive diagnosis can be established through joint aspiration and subsequent fluid analysis with cell count, gram stain, cultures, and the presence of needle-shaped urate crystals under polarized light microscopy. Elevations in complete blood count, ESR, and CRP levels can occur in both infection and gout [9,32]. Our patient suffered a postoperative periprosthetic infection following poor wound healing from discharging tophaceous material. It is possible that the source of tophaceous material could be an overlooked synovial deposit of urate crystals, mainly in the soft tissues, such as the patella tendon, which may have been disturbed by the surgical manipulation, although this would be unlikely due to the late onset and prolonged duration of the discharge; hence, de novo acute gouty arthritis is a more plausible explanation.

## Conclusions

This case report illustrates the complexity of treating knee osteoarthritis secondary to gout and the higher potential for complications. A semi-constrained knee replacement was used to compensate for the tibial bone loss secondary to gouty erosion and the potential for knee instability; a periprosthetic infection following poor wound healing from discharging tophaceous material was treated with DAIR. Prompt initiation of lifestyle modifications and medical treatment is crucial to prevent osteoarticular damage and avoid more complex surgical procedures.
